# Plant Antioxidant for Application in Food and Nutraceutical Industries

**DOI:** 10.3390/antiox8100453

**Published:** 2019-10-05

**Authors:** Monica Rosa Loizzo, Rosa Tundis

**Affiliations:** Department of Pharmacy, Health Science and Nutrition, University of Calabria, Via Pietro Bucci, 87036 Arcavacata di Rende (CS), Italy

Plants have been used since approximately 5000 BC not only for their medicinal properties, but also as food aroma and for their preservative activity [1]. Several classes of bioactive compounds including polyphenols, flavonoids, terpenes, and alkaloids have been identified [2]. 

Their health-promoting effects were largely demonstrated [3]. Moreover, phytochemicals can preserve food products by slowing down the autoxidation process, neutralizing free radicals, delaying the comparison of off-flavors, and inhibiting microbial growth [4,5,6]. The food oxidation process occurs when the food matrix is exposed to oxygen. Among food components, fats and oils or products rich in these components are more prone to the oxidation process. The oxidation implies the transfer of electrons from a substance to an oxidizing agent to generate free radicals that initiate chain reactions, leading to cell membrane, DNA, and protein damage as well as lipid peroxidation [7].

Phytochemicals characterized by antioxidant activity remove free radicals and inhibit oxidative reactions by being oxidized themselves [8]. Some antioxidant compounds (ubiquinol and glutathione) are produced in the body during normal metabolism. Other antioxidants (vitamins and phytochemicals) are assumed by the diet since fruits and vegetables are rich in these precious compounds.

Natural antioxidants have been found to be promising strategies to counteract the undesirable effects of oxidative stress. Oxidative stress has been identified as a major causative factor in the development and progression of several diseases, including cardiovascular and neurodegenerative diseases, as well as cancer [9]. In the last twenty years, several researches have been carried out to evaluate natural antioxidants for their potential use as additives in food products or to develop products such as functional foods and/or nutraceuticals. The use of nutraceutical products has been demonstrated to exert physiological benefits or provide protection against chronic diseases [10]. Another advantage is that the use of natural products does not require these compounds to be listed on the label, generating clean-label products that are looked for by consumers.

Scrob et al. [11] investigated the influence of gastrointestinal digestion of *Brassica oleracea* (broccoli) on its phytochemical content with particular reference to carotenoids, phenols, chlorophyll, and radical-scavenging activity. For this purpose, broccoli was extracted by ethanol at 30 °C for 20 min with ultrasound assistance. The assumption of carotenoids and chlorophyll content were considerably affected by gastric digestion whereas phenols were significantly decreased after intestinal digestion. This study confirmed that carotenoids are instable at low pH values (gastric conditions).

However, their bioaccessibility depends on the different types of carotenoids as well as on the food matrix in which these compounds are present, or how this food is cooked [12]. Regarding phenols, it is well known that these compounds are metabolized as consequence of oxidation or hydrolyzation processes. Moreover, they interact with dietary constituents such as proteins, fibers and lipids, changing their structure and affecting their bioavailability [13]. 

*Citrus limon* cv Femminello comune is a widely consumed Italian IGP (protected geographical indication) product. Its juice was subjected to ultrafiltration (UF) process using a cellulose acetate membrane in order to evaluate if this process enhances its healthy properties [14].

Physicochemical parameters, and total phenols and flavonoids contents were evaluated. The most abundant flavonoids, such as rutin, hesperidin, eriocitrin, and neohesperidin, were quantified.

The potential antioxidant and hypoglycemic activities were examined. UF juice obtained at 1.5 bar showed the highest antioxidant activity and exhibited promising α-amylase and α-glucosidase inhibitory activity. 

Terpenes represent another promising class of bioactive natural compounds. Bonesi et al. [15] studied the antioxidant and cholinesterase inhibitory activity of *Prunus armeniaca* and *P. domestica* leaves’ essential oils. All investigated samples were characterized by an interesting 2,2’-azino-bis(3-ethylbenzothiazoline-6-sulphonic acid) (ABTS) radical scavenging activity. *P. domestica* essential oils resulted as more active than *P. armeniaca* in inhibiting lipid peroxidation. A different behavior was observed in cholinesterase inhibitory activity. In fact, *P. armeniaca* oils were more active than *P. domestica* against acetylcholinesterase (AChE). 

Coffee is one of the beverages extensively consumed in the world. Roasted coffee beans are the most used, while coffee leaves are mainly used as infusion for traditional medicinal purposes in several countries. They showed some antioxidant compounds. Rodríguez-Gómez et al. [16] developed a liquid chromatography-electrochemical detection (LC-EC) method for the determination of three chlorogenic acid isomers, namely 3-, 4-, and 5-caffeoylquinic acids (CQA) in eight coffee leaf aqueous extracts (*Coffea anthonyi, C. arabica, C. canephora, C. charrieriana, C. liberica, C. liberica var. liberica, C. humilis,* and *C. mannii*). 5-CQA resulted to be the most abundant isomer, and some species contained a very low amount of CQAs. Fluctuations were observed depending on the *Coffea* species and harvesting period. Data suggested the preferential selection of *C. liberica* leaves collected in July due to the high total content in CQAs. 

Two capsaicin derivatives, namely *N*-docosahexaenoyl vanillylamine (DHVA) and *N*-eicosapentaenoyl vanillylamine (EPVA), were synthesized from their corresponding long chain polyunsaturated fatty acids, docosahexaenoic acid (DHA) and eicosapentaenoic acid (EPA) both important dietary components, and studied for their antioxidant and carbohydrateshydrolyzing enzymes’ (α-amylase and α-glucosidase) inhibitory activity together with capsaicin and the corresponding *n*-3 polyunsaturated fatty acids [17]. Both fatty acids showed a promising activity in comparison to the acarbose a largely prescribed drug for the treatment of hyperglycaemia. Structural changes in capsaicin derivatives had higher impacts on α-glucosidase than on α-amylase inhibition. Both derivatives are characterized by the absence of pungency and for this reason are characterized by a good compliance with respect to capsaicin. For the high bioactivity and pungency absence DHVA and EPVA could be used in formulation of functional foods useful in the management of type 2 diabetes and border-line hyperglycaemic patients.

Šalamon et al. [18] critically analyzed literature data about *N*-acetylcysteine (NAC), commonly found in onion. This compound is a precursor of glutathione. Actually, NAC is used in food supplements and cosmetics. Moreover, there are several uses in preclinical and clinical phases. Although NAC is considered safe, results from clinical trials are sometimes controversial or incomplete. 

*Rubus idaeus* (red raspberry) fruits are rich in antioxidants. One promising food industry application was described by Giuffrè et al. [19]. Red raspberry fruits were stored in different temperature conditions (1 °C and −20 °C) using new patented films namely nanoactive A and B, and polyethylene terephthalate. Both nanoactive A and polyethylene terephthalate prolonged and preserved the antioxidant properties of red raspberries compared to fruits stored with nanoactive B.

The fruits stored in the nanoactive film A showed better performance regarding storage in the fridge or freezer.

In conclusion, natural antioxidant use in food products can increase quality and add value. Therefore, novel environmentally friendly procedures of extraction, purification, identification and quantification of natural antioxidants from plants need to be developed to improve the extraction yields and market value of natural products.
